# The complete chloroplast genome sequence of the plant *Euonymus fortunei* (Celastraceae)

**DOI:** 10.1080/23802359.2018.1473724

**Published:** 2018-05-23

**Authors:** Wenping Hua, Chen Chen

**Affiliations:** a Department of Life Sciences, Shaanxi XueQian Normal University, Xi’an, Shaanxi Province, China;; b Xi’an Botanical Garden of Shaanxi Province, Institute of Botany of Shaanxi Province, Xi’an, Shaanxi Province, China

**Keywords:** *Euonymus fortunei*, Illumina sequencing, chloroplast genome

## Abstract

Chloroplast (cp) genome sequences become a useful popular tool for population and phylogeny in recent reports. Here, the complete cp genome of the *Euonymus fortunei* has been reconstructed from the whole-genome Illumina sequencing data. The circular genome is 157,639 bp in size, and comprises a pair of inverted repeat (IR) regions of 26,668 bp each, a large single copy (LSC) region of 85,956 bp and a small single copy (SSC) region of 18,347 bp. The total GC content is 37.2%, while the corresponding values of the LSC, SSC, and IR region are 35.1%, 31.7%, and 42.7%, respectively. The cp genome contains 126 genes, including 89 protein-coding genes, eight ribosomal RNA genes, and 29 transfer RNA genes and one pseudogene. The maximum-likelihood phylogenetic analysis showed a strong sister relationship with *Euonymus japonicus* in Celastraceae. Our findings provide a foundation for further investigation of cp genome evolution in *E. fortunei* and other higher plants.

The chloroplast (cp) contains its autonomously replicating DNA genome that proceeds with photosynthesis and many other biological activities such as synthesizing starch, fatty acids, and other proteins relative to its special functions (Ohyama et al. 1986; Bausher et al. 2006). Most cp genomes are circular DNA molecules which have the typical quadripartite structure containing two repeat regions (IRa and IRb), LSC, and SSC (Yang et al. 2010).

*Euonymus fortunei* (Celastraceae), an evergreen shrub, is native to East Asia including China, Korea, and Japan. As an ornamental plant, *E. fortunei* has a wide range of applications in vertical garden and soil and water conservation. In addition, *E. fortunei* also serves as a traditional medicine for the treatment of inflammation and neurological diseases (Jian 2007; Zuo et al. 2012). To facilitate its genetic studies and thus contribute to its development and sustainable utilization, we assembled its cp genome using high-throughput Illumina sequencing technology in this study, as well as analysed its phylogenetic evolution, which will be helpful for better understanding of evolution within the Celastraceae and further studies on its molecular breeding and genetic engineering.

DNA extraction form the fresh leaves was collected from a single individual of *E. fortunei* in Xi’an Botanical Garden (Xi’an, Shaanxi Province, China). High-throughput DNA sequencing was conducted on the Illumina HiSeq 2500 Sequencing System (Illumina, San Diego, CA) by Breeding Biotechnologies (Breeding, Yangling, China). Total 16.8 M 150 bp raw reads were retrieved and trimmed by CLC Genomics Workbench v8.0 (CLC Bio, Aarhus, Denmark). A subset of 12.6 M trimmed reads were used for reconstructing the cp genome by NOVOPlasty (Dierckxsens et al. 2016), with that of its congener *Euonymus schensianus* (GenBank: NC_036019.1) as the initial reference genome. A total of 23,125,435 individual cp reads yielded an average coverage of 594.8-fold. The cp genome was annotated in GENEIOUS R9 (Biomatters Ltd., Auckland, New Zealand) by aligning with that of *E. schensianus* (NC_036019.1) and was drawn to the circular cp genome sequence map of OGDRAW 1.1.

The cp genome of *E. fortunei* is a double-stranded circular DNA molecule with 157,639 bp in size (MH150885). It comprises a pair of inverted repeat (IR) regions of 26,668 bp each, separated by a large single copy (LSC) region of 85,956 bp and a small single copy (SSC) region of 18,347 bp. The total GC content is 37.2%, while the corresponding values of the LSC, SSC, and IR region are 35.1%, 31.7%, and 42.7%, respectively.

This cp genome harbours 126 functional genes, including 89 protein-coding genes (PCGs), 29 tRNA genes, and eight rRNA genes. Eighteen PCGs, eight tRNA genes, and all rRNA genes are duplicated in the IR regions. The LSC region possesses 59 PCGs and 20 tRNA genes, while the SSC region contains 12 PCGs and one tRNA gene. Thirty three PCGs, 14 tRNA genes, and four rRNA genes are located in the forward strand while others are located in the reverse strand. Moreover, 14 genes contain one intron, while *ycf3* harbours two introns; all the other genes are intronless. Among those genes, 44 are involved in photosynthesis, and 53 genes are involved in self replication. This is similar to those previously reported for the cp genomes of most other vascular plants (Yang et al. 2014).

Sixty PCGs data among 41 cp sequences were aligned by MAFFT (Katoh et al. 2002) and then were connected as gene strings. The maximum-likelihood phylogenetic tree of *E. fortunei* was generated using those gene strings sequence by MEGA 6.0 (Tamura et al. 2013) with using 500 bootstrap replicates (Figure 1). The phylogenetic analysis showed the position of *E. fortunei* was situated as the sister of *E. japonicus* and *E. schensianus* in Celastraceae. Our findings provide a foundation for further investigation of cp genome evolution in *E. fortunei* and other higher plants.

**Figure 1.**
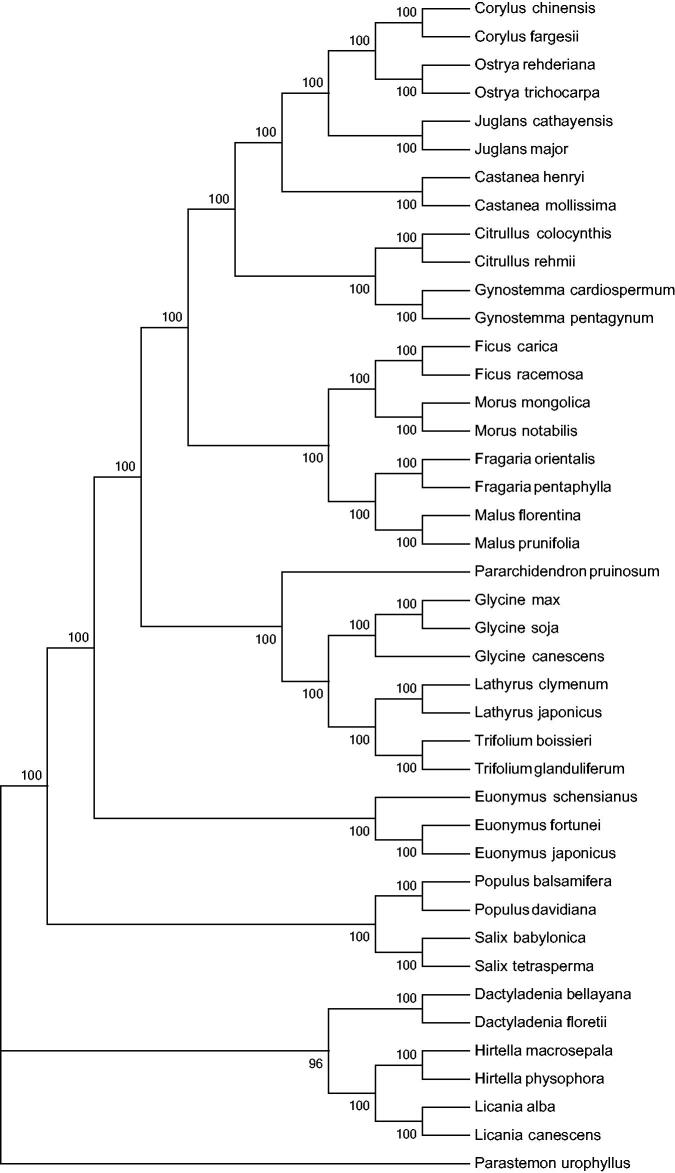
Phylogenetic data of 41 species within the family Celastraceae based on the maximum-likelihood analysis of the whole chloroplast genome sequences using 500 bootstrap replicates. The analysed species and corresponding GenBank accession numbers are as follows: *Corylus chinensis* NC_032351.1, *Corylus fargesii* NC_031854.1, *Castanea henryi* NC_033881.1, *Castanea mollissima* NC_014674.1, *Citrullus colocynthis* NC_035727.1, *Citrullus rehmii* NC_035975.1, *Dactyladenia bellayana* NC_030555.1, *Dactyladenia floretii* NC_030557.1, *Euonymus japonicus* NC_028067.1, *Euonymus schensianus* NC_036019.1, *Fragaria orientalis* NC_035501.1, *Fragaria pentaphylla* NC_034347.1, *Ficus carica* NC_035237.1, *Ficus racemosa* NC_028185.1, *Glycine canescens* NC_021647.1, *Glycine max* NC_007942.1, *Glycine soja* NC_022868.1, *Gynostemma cardiospermum* NC_035959.1, *Gynostemma pentagynum* NC_036136.1, *Hirtella macrosepala* NC_030561.1, *Hirtella physophora* NC_024066.1, *Juglans cathayensis* NC_033893.1, *Juglans major* NC_035966.1, *Juglans cathayensis* NC_033893.1, *Juglans major* NC_035966.1, *Lathyrus clymenum* NC_027148.1, *Lathyrus japonicus* NC_027075.1, *Licania alba* NC_024064.1, *Licania canescens* NC_030566.1, *Malus florentina* NC_035625.1, *Malus prunifolia* NC_031163.1, *Morus mongolica* NC_025772.2, *Morus notabilis* NC_027110.1, *Ostrya rehderiana* NC_028349.1, *Ostrya trichocarpa* NC_034295.1, *Pararchidendron pruinosum* NC_035348.1, *Parastemon urophyllus* NC_030517.1, *Populus balsamifera* NC_024735.1, *Populus davidiana* NC_032717.1, *Salix babylonica* NC_028350.1, *Salix tetrasperma* NC_035744.1, *Trifolium boissieri* NC_025743.1, and *Trifolium glanduliferum* NC_025744.1.
